# Updates on Dengue Vaccine and Antiviral: Where Are We Heading?

**DOI:** 10.3390/molecules26226768

**Published:** 2021-11-09

**Authors:** Harun Norshidah, Ramachandran Vignesh, Ngit Shin Lai

**Affiliations:** 1 Institute for Research in Molecular Medicine (INFORMM), Universiti Sains Malaysia, Gelugor 11800, Penang, Malaysia; norshidahharun@gmail.com; 2 Faculty of Pharmacy and Health Sciences, Universiti Kuala Lumpur-Royal College of Medicine Perak, Ipoh 30450, Perak, Malaysia; 3 Faculty of Medicine, Universiti Kuala Lumpur-Royal College of Medicine Perak, Ipoh 30450, Perak, Malaysia; vignesh@unikl.edu.my

**Keywords:** dengue virus, NS2B/NS3pro, antiviral, drug discovery, vaccine

## Abstract

Approximately 100–400 million people from more than 100 countries in the tropical and subtropical world are affected by dengue infections. Recent scientific breakthroughs have brought new insights into novel strategies for the production of dengue antivirals and vaccines. The search for specific dengue inhibitors is expanding, and the mechanisms for evaluating the efficacy of novel drugs are currently established, allowing for expedited translation into human trials. Furthermore, in the aftermath of the only FDA-approved vaccine, Dengvaxia, a safer and more effective dengue vaccine candidate is making its way through the clinical trials. Until an effective antiviral therapy and licensed vaccine are available, disease monitoring and vector population control will be the mainstays of dengue prevention. In this article, we highlighted recent advances made in the perspectives of efforts made recently, in dengue vaccine development and dengue antiviral drug.

## 1. Introduction

Dengue virus (DENV) one of the human arboviruses from the *Flaviviridae* family, places a significant impact amongst 125 tropical and subtropical regions. Approximately 390 million infections affect the global population annually [1]. Out of the 390 million cases, 500,000 to 1,000,000 infections are severe cases that lead to fatalities. Endemicity is observed in more than 100 countries, including Africa, the Eastern Mediterranean, the Americas, Southeast Asia, and the Western Pacific. The latter three are the most severely afflicted, with Asia accounting for 70% of the worldwide illness load [2]. Large scale dengue outbreaks occurred in several countries in the recent past, including the 2019 epidemics in Nepal [3] the 2019 outbreaks in Dhaka, Bangladesh [4] also including the unexpected massive outbreaks in Xishuangbanna (a border area of China), Myanmar, and Laos in 2019 [5]. According to a prediction model developed by Messina, J.P. et al., 2019, the number of people infected with dengue would grow by 2.25 billion between 2015 and 2080 [6]. In addition to these global trends, rising temperatures attributed to climate change have raised concerns that dengue will worsen in already endemic areas due to faster viral amplification, increased vector survival, reproduction, and biting rate, ultimately leading to longer transmission seasons and a greater number of human infections, with a greater number of severe infections expected. Temperature rises may worsen the problem by allowing for increased dissemination and transmission in low-risk or currently dengue-free areas of Asia, Europe, North America, and Australia. Hence, the World Health Organization (WHO) has recently announced that dengue infection is one of the top ten most significant threat to global health in 2019.

DENV is an enveloped virus with icosahedral symmetry and a genomic size of around 11 kb [7]. It has a positive single-stranded RNA genome that encodes for a single open reading frame and can be translated into three structural proteins, the core (C), premembrane/membrane (prM/M), and envelope (E), as well as seven non-structural (NS) proteins, namely NS1, NS2a, NS2b, NS3, NS4a, NS4b, and NS5 [8]. Its structural glycoprotein E is in charge of cell identification and encouraging host entrance, which is accomplished by a fusion process between the viral envelope and the cell membrane, while the NS protein aids viral genome replication [9].

**Figure 1:**
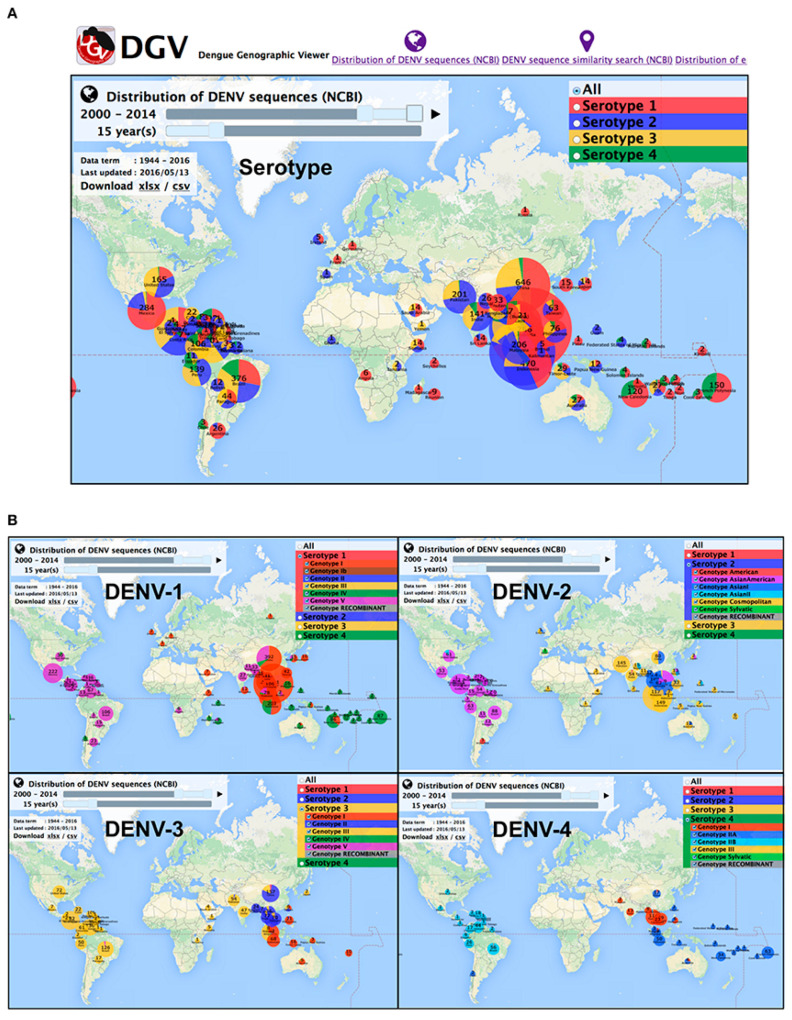
Overview of DENV serotypes and genotype global distribution in 2000–2014. (**A**) DENV serotypes distribution (**B**) DEN genotypes distribution for each serotype. Source: Yamashita et al., 2016 [12].

DENV is spread to humans by female Aedes mosquitos in four different serotypes (DENV1–4). The four serotypes are further subdivided into phylogenetic groups, each with its unique genotype. The icosahedral viral genome, which expresses itself as the DENV1–4 serotypes with 65–70% sequence identity, is the taxonomically distinguishing component [10]. The genome sequence categorizes serotypes into different lineages with high genetic diversity [7]. The regional assimilation of viral serotypes and genotypes from local geographical proximity, as well as their extensive dispersion, can lead to regional population movement and trans-border economic activity [11]. Furthermore, viral genotypes may differ dependent on geographical distribution, epidemic potency, and other factors. To assist the tracing of DENV outbreak isolates and aiding the control of the infection, Yamashita, A. and colleagues presented a comprehensive database of DENV sequences containing both serotype and genotype data together with the epidemiological data (Figure 1) [12].

**Figure 2:**
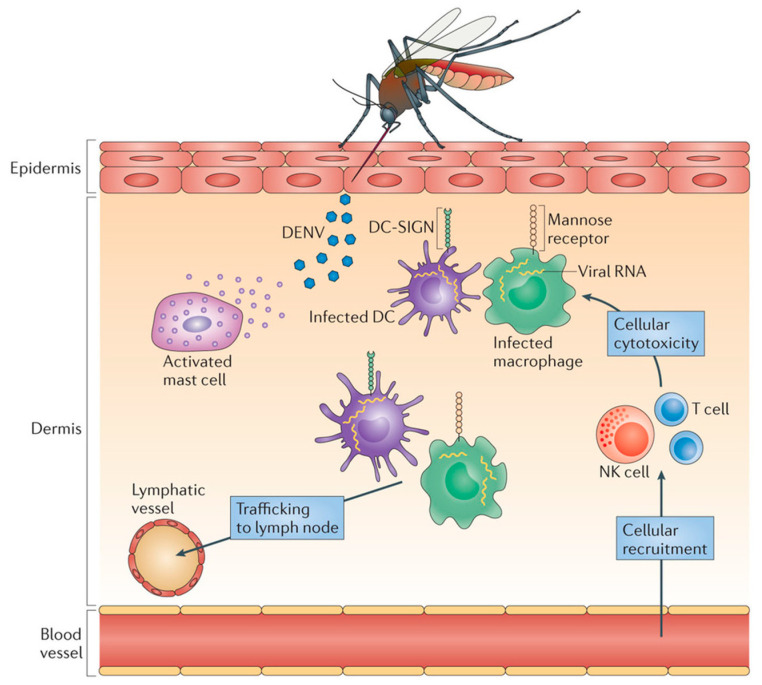
Dengue pathogenesis. Released viral particles may infect adjacent cells (mostly monocytes or dendritic cells (DCs)) or trigger local immune cells. A local inflammatory response to DENV in the skin induces the recruitment of vasculature-derived leukocytes, including natural killer (NK) cells and T cells, which enhance the death of virus-infected cells at the injection site. DENV is then expected to spread to draining lymph nodes through lymphatic channels, causing systemic infection. These localized inflammatory reactions occur several days before any symptoms appear. Adapted with permission from ref. [7]. Copyright 2013, St. John, A. et al.

During the infection, the virus enters the body and infiltrates local macrophages, and multiplies. Locally infected cells then move from the site of infection to lymph nodes, where monocytes and macrophages are recruited and become infection targets. As a result, the infection multiplies and the virus spreads via the lymphatic system. During this primary viremia which manifests itself within 24 h, many mononuclear cells, including blood-derived monocytes, were activated. Bone marrow cells have also been found to be vulnerable to DENV infection. The viral load is quite high in severe cases, and many critical organs are impacted. Infected macrophages generate a variety of signaling proteins, including interferons, cytokines, chemokines, TNF, and other mediators, which are responsible for a variety of symptoms. Consequently, these mediators have an effect on the body’s hemostatic system. Fluid begins to leak from blood vessels, causing blood volume to drop and resulting in low blood pressure and inadequate supply of oxygen to critical organs such as the brain. Dengue also affects bone marrow, preventing it from producing enough platelets resulting in blood clotting deficiency and increasing the risk of bleeding (Figure 2).

Dengue infection can be asymptomatic and symptomatic in some infected persons. The common clinical symptoms experienced by an infected person include headaches, fever, fatigue, urticaria, body aching, lymphitis, and leukocytopenia. Dengue hemorrhagic fever (DHF) develops in severe instances and is characterized by hemostasis irregularities and increased vascular permeability, the latter of which can lead to hypovolemic shock (dengue shock syndrome, DSS) [13]. In a person who has not previously been infected by any flavivirus, known as primary infection, the ratio of IgM and IgG is high. Whilst in secondary infection, the host is immunologically sensitized to dengue or other flavivirus infection and the ratio of both immunoglobulins is low.

Despite the lack of specific antiviral therapy and contentious approved vaccine by the US Food and Drug Administration (FDA), dengue maintains as a major worry in 129 endemic countries. Hence, dengue prevention continues to rely on disease surveillance and vector population control [14,15]. This review focuses on the current status and challenges of DENV antiviral and vaccines development, hence shedding some light on our direction in overcoming dengue disease.

## 2. Dengue Vaccine Development

**Figure 3:**
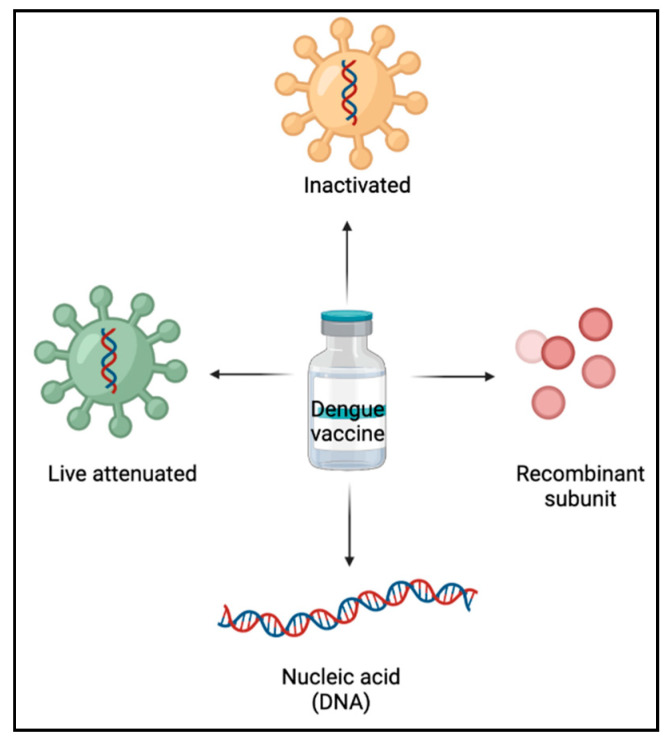
Types of dengue vaccines.

In the development of dengue vaccines, a thorough understanding of immune responses to DENV aid in the formulation of an effective approach [16]. Live-attenuated vaccines, inactivated vaccines, recombinant subunit vaccines, and nucleic acid (DNA) vaccines are the primary forms of dengue vaccines currently under research (Figure 3).

These types of vaccines confer protection by increasing the immune responses to the E protein and non-structural protein 1 of the dengue virus (DENV) (NS1). The vaccine candidates that have progressed to the clinical trial stage are summarized in Table 1 below.

### 2.1. Live-Attenuated Vaccines

Antigenic compounds synthesized from a living pathogen that has been engineered to be less virulent or avirulent are known as live-attenuated vaccines. Once administered, the viruses multiply locally, eliciting neutralizing antibodies and cell-mediated immune responses against the four dengue virus serotypes. These vaccines demonstrate the benefits of delivering protective antigens while offering long-term immune protection [31]. Using recombinant DNA technology, several live dengue attenuated vaccines have been developed.

Dengvaxia, also known as CYD-TDV, is the only approved tetravalent live-attenuated dengue vaccine candidate [11,17,18]. Receiving FDA approval in 2015, this vaccine is now accessible in more than 20 countries. Its usage has been allowed with strict restrictions on the recipients’ age and serostatus [32]. It offers high effectiveness in preventing dengue disease caused by DENV serotypes 1–4 and is safe in people who have had a past dengue infection, i.e., those who are seropositive. To date, the FDA has approved the usage in individuals 9 through 16 years of age with laboratory-confirmed previous dengue infection and living in endemic areas [33]. However, for those who are seronegative, the vaccine increases the chance of having severe dengue when the person has a spontaneous dengue illness about 3 years following immunization. Vaccination in the naive subjects stimulates the development of neutralizing antibodies against all four DENV serotypes. Specific antibodies against one or a few serotypes dominate this response, whereas reactions against the other serotypes are mostly due to cross-reactive antibodies. Moreover, it produces serotype-specific and cross-reactive T cell responses against DENV structural antigens. Therefore, the seronegative individuals may thus constitute a subclinical attenuated ‘primary-like’ illness. Vaccination in this group also results in different immunological effects depending on the serotypes. When compared to cross-protection evoked by vaccination in seropositive individuals, minimal cross-protection in seronegative individuals can be observed, posing a greater risk of inducing antibody-dependent enhancement (ADE) [34,35,36]. Consequently, WHO recommends that this vaccine is only given to seropositive individuals.

Tetravax (TV003/TV005), on the other hand, differs significantly from CYD-TDV in terms of the viral particle structure, infectivity, and immunogenicity [20,37]. To reduce DENV virulence, researchers utilized three untranslated regions (UTRs) deletions and structural gene prM/E chimerization. In comparison to TV003/TV005, CYD had a greater risk of viremia, lesser dengue virus type 2 resistance, and a reduced level of adaptive immune response [37].

TAK-003, often known as DENVax, is a live-attenuated chimeric tetravalent dengue vaccine [21]. At present, it is still in phase III of clinical investigations. The backbone of this vaccine is a weakened DENV2 strain (PDK-53) that contains the prM/E parts of all serotypes. The vaccine was proven to be immunogenic and well-tolerated in multiple phase I and II clinical studies, independent of the participants’ age or serostatus. TAK-003 showed a DENV serotype-dependent protective effectiveness, similar to its predecessor Dengvaxia, but with higher levels of DENV2 neutralizing antibodies and lower DENV3 and DENV4 protection rates, consequently, its safety profile is not entirely known [38]. Although a previous clinical study indicated it triggers CD8+ T lymphocytes directed at NS1, NS3, and NS5 in patients that have never been infected with DENV [39], this data were not included in later clinical studies.

In addition to live-attenuated dengue vaccines is TDEN F17/F19 [22,23]. In a phase II trial, this vaccine was proven to be a safe, well-tolerated, and immunogenic DENV vaccine candidate. One month following the second dosage, antibody responses to all four DENV types were recorded in more than half of the infants/toddlers and all of the children [40]. The vaccines used in these studies were lyophilized monovalent vaccines that were combined into a tetravalent vaccine at the point of administration.

Conclusively, based on the experiences obtained during the development prospective live-attenuated vaccines, the WHO have highlighted the guidelines on the quality, safety, and efficacy of the dengue tetravalent vaccines (live, attenuated). Furthermore, according to FDA, dengue live-attenuated vaccine, particularly Dengvaxia, elicits dengue-specific immune responses against the four dengue virus serotypes after injection. However, the exact mechanism of protection has yet to be discovered [33].

### 2.2. Inactivated Vaccines

Inactivated vaccines are antigenic compounds made up of denatured substances from other microbes such as bacteria and viruses that can provide protection against the live pathogen [41]. This vaccine stimulates immunity by using antigens from the capsid (c), membrane (M), envelope (E), and non-structural 1 (NS1) protein, although composite vaccinations provide superior protection compared to single-type immunizations.

The tetravalent purified formalin-inactivated virus (TPIV), which contains four non-active dengue serotypes, is an example of an inactivated vaccine now in clinical trials [42]. TDENV PIV/AS03B is now being investigated in a clinical study with various dosing regimens [24,25]. In both flavivirus-naive and experienced groups of infected individuals, TDEV PIV was well tolerated and immunogenic. Furthermore, this type of vaccine is safer than live-attenuated vaccines since there is no risk of reactivation and immunological balance is better regulated.

### 2.3. Recombinant Subunit Vaccine

Following the failure and controversy surrounding Dengvaxia^®^, recombinant subunit vaccination options have regained some interest. In this type of immunization, antigenic proteins produced by prokaryotic or eukaryotic cells generate long-lasting protective/therapeutic immune responses [43]. By far the most prevalent DENV recombinant subunit candidates are the envelope E protein or shortened variants. These rely on the formation of neutralizing antibodies to prevent DENV from infecting its host cells.

Despite the ease with which recombinant dengue proteins may be expressed in *E. coli*, there are potential concerns of being exposed to endotoxin contaminants and protein misfolding [44]. V180, which is made up of a shortened version of the protein DEN-80, is the most promising subunit vaccine [26,27]. Recombinant subunit vaccinations are more likely than live-attenuated vaccinations to induce steady immune responses against all DENV serotypes, decreasing the likelihood of the ADE effect [35].

### 2.4. DNA Vaccine

In the development of DNA vaccines, no less than a gene encoding specific antigens were incorporated into the plasmid. The E glycoprotein anchored to the prM protein mediates the first interaction between DENV and host cells, hence, it becomes the primary target for inducing neutralizing antibodies. Through in vivo injection of the expressed antigens, the vaccines induce both arms of the immune system which are the T cell responses and antibody production [45].

D1ME100 is an example of a DNA vaccine that has been clinically tested on *Aotus nancymaae* monkeys and humans [30,46]. In the initial phase of immunization, the vaccine proved to be safe and tolerable. Notwithstanding, with only about half of those who had high-dose immunization producing neutralizing antibodies, this immunogenicity produced was found to be weak. Furthermore, in those who received low-dose immunization, no neutralizing antibody response was detected [28]. Another example is the tetravalent dengue vaccine (TVDV), which is made up of four plasmids carrying the prM and E encoding genes from each DENV serotype [28,29]. The robust DENV-specific IFN T-cell response generated in 79% of the highest vaccination dosage recipients [29], however, is cause for concern. Despite its lack of immunogenicity, this vaccine has been shown to be stable, simple to make, inexpensive, and large-scale production. Plasmid modification with extremely efficient promoters, alternative delivery strategies, multiple doses, and co-immunization with adjuvants are recommended as solutions to the drawbacks of this form of vaccination.

### 2.5. An Ideal Vaccine against Dengue

Despite the fact that a dengue vaccine is now available, which is an important step forward, the long-lasting protective efficacy against each of the four dengue virus serotypes has yet to be confirmed. The characteristics of DENV, as well as the immunological protection and pathologic processes involved, including the transmission and epidemiology of dengue illness, have all hindered the development of a dengue vaccine. Vaccine development also has encountered conceptual problems since its inception, including the biology of the viruses that cause it, immunological protection and pathogenesis processes, and transmission and epidemiology.

Moreover, in endemic regions, the establishment of a protective antibody response against the infection is acquired through recurrent viral exposure. After being infected with one of the four DENV serotypes, the patient can be infected with any of the other serotypes. However, the infected individuals most likely do not manifest the clinical symptoms as a consequence of cross-protection.

The ideal dengue vaccine is abridged from the fact that it must elicit a multitypic response similar to that of people living in endemic areas, aided by persistent exposure and perhaps symptomatic or asymptomatic reinfection. An explicit elaboration on factors influencing vaccine responses, such as the pre-vaccination environment, as well as the significant challenges that face the development of an efficient/protective dengue vaccine, such as the presence of multiple serotypes, ADE, and cross-reactivity with other flaviviruses, have also been discussed to enhance the search of reliable dengue vaccine [47]. Hence, we concluded the key barriers to dengue vaccine development include a lack of a proper mechanistic investigation in pathogenesis and ADE.

## 3. Dengue Antiviral Drug Discovery

As a consequence of the high prevalence of dengue fever and the lack of a widely applicable vaccine, an efficient antiviral agent to treat DENV infection is urgently needed. By and large, there are two approaches to antiviral drug discovery. Based on the inhibitory mechanism, the approaches include inhibitors that target host cell factors and inhibitors that target viral components known as direct-acting antivirals (DAAs). Each method has advantages and disadvantages. A host targeting approach enables a broad spectrum of action while avoiding drug resistance due to a greater genetic barrier. However, since the drug targets host components involved in certain cellular activities, the risk of toxicity and adverse effects is high. Due to the lack of a predictive in vitro model, this strategy has a disadvantage in establishing a correlation between in vitro and in vivo methods. On the other hand, DAAs are a promising method since they directly target a viral protein, consequently, exert low toxicity and a broad therapy window. However, one well-known disadvantage of DAA is the relatively high possibility of resistance development [10]. In spite of the demerits, this approach is used by the majority of authorized antiviral agents and those in clinical trials. Hence, in this review, we will give a perspective on current DAAs in dengue treatment focusing on the target proteins, mechanism of action, and its current status and challenges.

**Figure 4:**
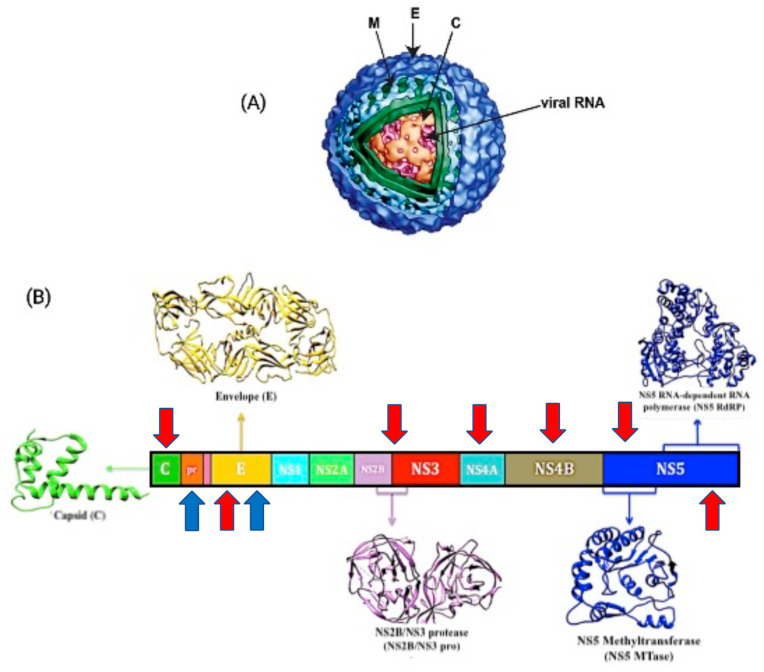
Structure and genome of the dengue virus. (**A**) Diagram of structural proteins dengue virus particles. M: membrane, E: envelope, C: capsid. (**B**) Diagram of the flavivirus genome organization and expression: structural (S) and nonstructural (NS) proteins. Dengue vaccine target antigens and antiviral target proteins discussed in this review are marked with blue and red arrow, respectively. (Figure modified from Nncube, N.B. et al., 2018).

There are several crucial points in the life cycle of DENV where the antiviral treatment can be used to efficiently limit virus replication and lessen the viral load. Resembling the main purpose of dengue vaccines, its effectiveness against all four serotypes is decisive. As viremia levels increase within 24 to 48 h, it is requisite for the antiviral to act promptly. Aiming at the viral enzymes has been the most promising strategy in developing dengue antiviral candidates. The discovery of novel antiviral drugs that target viral entry, RNA replication, and polyprotein cleavage could help to prevent dengue infection [48,49]. Few antiviral candidates and their specific therapeutic targets have been explored but their biological effects are yet to be disclosed. The DENV E protein, C protein, NS2B/NS3 protease, NS5 RNA-dependent RNA polymerase (RdRp), NS5 methyltransferase (MTase), NS4A, and NS4B have all been recognized as potential targets for antiviral medication development in the last five years. Here, we illustrate the antiviral target sides in Figure 4 and summarize the recent potent inhibitors and their potential DENV targets mechanisms in Table 2.

### 3.1. The Antiviral Targets

#### 3.1.1. E Protein

The envelope glycoprotein of DENV, also known as E protein, is a promising therapeutic target since it is necessary for the attachment and fusion of the DENV into the host cells. The advantage of targeting the E protein is that it allows the drugs to interact extracellularly, obviating the requirement for membrane-permeable inhibitors. Furthermore, due to its non-effect on the intracellular proteins, impermeable drugs that target the E protein extracellularly may have minimal toxicity.

The E protein domain III has been identified as a possible target for preventing viral entrance into the cell [82]. Based on the external loop of domain III, DET4, a synthetic peptide was designed and its van der Waals and electrostatic interactions were studied through an in silico approach [51]. Moreover, as the external loop is a conserved region across all dengue serotypes, its possibility towards all four serotypes is assertive. Another viral entry inhibitor that was explored through computer simulation was MLH40. This synthetic peptide resembles the ectodomain region of the DENV that is preserved and exhibits significant inhibition activity by blocking 80% of DENV attachment to the various cell types [50]. In addition, in silico analysis revealed that its N-terminal loop interacts with DENV E proteins, causing them to dimerize. Besides synthetic peptide, a small molecule, BP34610, also displayed an inhibitory effect on all four dengue serotypes by targeting the E protein. Besides being capable of affecting the early stage of viral entry, this inhibitor portrays a synergistic effect with ribavirin against DENV [52].

#### 3.1.2. C Protein

The first protein encoded in the viral genome is the capsid (C) protein, which is followed by prM. It is a homodimer protein with a disorderly N-terminus, an intermediate malleable fold portion, and a substantially conserved fold region.

VGTI-A3-03, a recent drug that suppresses C protein, works by directly attaching to the dengue viral capsid protein and incorporating it into the viral particle [54]. Moreover, from the findings of the recent DENV capsid protein-peptide inhibitor, Pep14–23, it is proposed that when the C protein and the system of the host lipid interact, minimal allosteric alterations that can modify the specific binding of the protein to viral RNA [53].

The structure of the DENV capsid protein, its subcellular localization during infection, and its interaction with host components have all been explored. Nonetheless, there are a number of critical concerns that have yet to be explored. It is vital to know how the capsid recruits the viral DNA to assemble nucleocapsids, as well as how viral and host proteins play a role. Furthermore, more research is needed on the functional importance and dynamics of capsid accumulation on lipid droplets and nucleoli.

#### 3.1.3. NS2B/NS3 Protease

One of the most well-studied targets for the development of anti-infective therapies against the dengue virus is the NS2B/NS3 protease. Owing to the multifunction of NS3 protein, the enzyme holds the activities of protease, helicase, and RNA triphosphatase. Flavivirus proteases, particularly NS2B/NS3, are clearly required for viral replication and infectivity.

In developing potent NS3pro/NS2B protease inhibitors, high-throughput screening (HTS) of small chemical libraries or peptidomimetics development that can imitate the natural substrate were utilized. Based on its structure, the flat active site of NS3pro led to challenges in designing potent inhibitors albeit with the assistance of structure-based design [83]. Since the preferred peptide substrate contains multiple positively charged amino acids, it is challenging for drug designers to construct peptides that inhibit DENV protease due to its non-oral bioavailability. In the last five years, work on small-molecule NS3pro/NS2B protease inhibitors has expanded, using HTS, examining natural sources, synthesizing rational drug design, and virtual assessment utilizing computer-aided drug design (CADD).

Most of all recent potent inhibitors of dengue NS2B/NS3pro target reported in Table 2 have employed combination methods in their work [55,56,57,58,59,60,61,84,85]. The majority of them have successfully established a good binding affinity outcome with the protease catalytic triad, namely His51, Asp75, and Ser135. Interestingly, different from other candidates, policresulen, an organic acid with hemostatic and antimicrobial activities, has shown that it directly interacts with Gln106 and Arg133 of the protease via hydrogen bonding. Furthermore, the in silico findings of policresulen together with nelfinavir, MB21, diaryl (thio)ethers, Compound **104**, carnosine have been supported with promising antiviral effects on virus cell-based assays. By directing the drugs to the NS2B/NS3 proenzyme, several of the candidates portray potential activity towards more than one DENV serotype [56,84] and amazingly also against other flaviviruses such as West Nile virus [59,61] and Zika virus [62,73].

#### 3.1.4. NS4A and NS4B

The NS4A and NS4B proteins are involved in virus replication and the interaction of DENV and host in a variety of ways. They interact with a Kd (dissociation constant) estimated to be 50 nM. Explanation on the characterization of both proteins are explicitly described by Zou, J., et al., 2015, however, to date, there are no crystal structures for NS4A and NS4B that have been reported [86].

The extremely hydrophobic protein NS4A, which induces membrane rearrangements, is necessary for the replication of DENV. Recently, it was revealed that NS4A promotes autophagy in epithelial cells, therefore, preserving the host cell from apoptosis. Nobori et al. (2018) demonstrated that Compound-**B**, with a benzimidazole structure, displayed antiviral activity specific to four DENV serotypes, through a DENV-infected cell-based assay [74].

NS4B also appears to have two hydrophilic segments and is likely to interfere with STAT1 phosphorylation, hence inhibiting the IFN-/-induced signal transduction cascade. Candidates that have been studied targeting this highly hydrophobic membrane protein are AM404 and Compound **14a**. AM404 is a paracetamol metabolite that exhibits anti-DENV activity, and NS4B mutations render the virus insensitive to AM404; the latter is a spiropyrazolopyridones compound that is orally available and has acceptable in vivo pharmacokinetic characteristics [75,87,88]. NITD-688, a pan-serotype preclinical candidate, has also demonstrated excellent oral efficacy features with a more promising in vitro safety profile [76].

#### 3.1.5. NS5 Protein: Methyltransferase (MTase) and Polymerase

The flavivirus nonstructural protein 5 (NS5) is the most conserved of the viral proteins. It has a molecular weight of approximately 900 kDa and contains enzyme activity that is essential for viral replication. Its N-terminal domain encodes dual N7 and 2′-O methyltransferase (MTase) activities, as well as presumably guanylyltransferase (GTase) enzymes involved in RNA cap formation. The C-terminus contains an RNA-dependent RNA polymerase (RdRp), which makes it essential for viral RNA synthesis [89]. Furthermore, dengue virus NS5 MTase and RdRp activities have been well studied, both structurally and functionally.

One of the recent inhibitors that exhibited significant anti-DENV activity is 2′-*C*-methylcytidine (2CMC) [77]. The employment of DENV replicon and infectious systems have proven the reaction of the antiviral nucleoside/nucleotide analog on viral replication. On the other hand, the NS5 MTase enzyme portrays some selectivity challenge due to its existence in humans. However, Vernekar et al. (2015) have proven that 3′-azidothymidine (AZT)-derived 5′-silylated nucleoside scaffold is constantly selectable inhibiting DENV at minimal micromolar concentrations [78]. Molecular modeling and docking have also revealed that the antiviral property of this compound is possible to bind to the MTase. The selectivity issue of the particular target has also been proven in a study by Bullard and colleagues where a lead compound known as BG-323 showed a high affinity for plasma binding proteins in humans [79]. Additionally, inhibition of both N7 and 2′-O MTase functions of several flavivirus MTases was likewise displayed by another promising anti-DENV candidate, NSC 12,155 [81]. The crystal structure of the MTase–inhibitor complex confirms the mechanisms and suggests avenues for future improvement as flavivirus MTase inhibitors [90].

### 3.2. Antiviral Origins

In addition to describing the potential antiviral candidates, we would also like to highlight the origins of the candidates. The small molecule or non-peptide candidates addressed in this review originate from either synthetic or natural sources. Currently, the majority of drugs used in clinical practice are synthetically produced. The preparation includes both chemical procedures (reaction) and phytochemical methods. The advantages of synthetic drugs are their chemical purity, ease of manufacture, and excellent quality. As a result, by changing the chemical structure of the prototype drug, more effective and safer pharmaceuticals can be created.

The utilization of natural sources, on the other hand, is a well-established strategy for identifying novel chemicals with potential therapeutic effects. This class of medications includes novel bioactive chemicals that are required for the manufacture of current medicines [90]. It also delivers many classes of novel bioactive lead molecules for drug discovery and development. Recent research has concentrated on bioactive compounds found in plants such as *Carica papaya* [65,67], *Ganoderma lucidum* var. antler [66], *Curcuma longa* [68], *Endiandra kingiana* [69], *Dryobalanops aromatic* [70], *Acorus tatarinowii Schott* [71], and *Syzygium aromaticum* [72]. In these studies, researchers included the extraction phase of crude plants in solvents, mostly methanol, before moving on to the subsequent tests to see how effective it was against DENV NS2B/NS3 protease. Nonetheless, based on our findings, both cohorts exert significant inhibitors with promising inhibitory efficacy against the dengue proteins, with the potential to advance to the next anti-DENV research step.

### 3.3. Future Perspective

Given the substantial number of potential compounds that have antiviral activity against DENV in vitro, only a few have been tested in animal models and humans. Additionally, the lack of a viable animal model of DENV infection, as well as the lengthy and costly process of discovering novel compounds, impede dengue drug research efforts. Hence, downstream research required for clinical translation of any therapeutic should be actively explored. The effort might shed some light on providing a clear and conclusive efficacy signal that will guide the design of larger trials. Moreover, such a strategy would avoid the need for lengthy and costly clinical studies, which frequently end in failure owing to insufficiently robust effectiveness.

## 4. Conclusions

In the past five years, several potential vaccine candidates and antiviral agents with anti-DENV activity have been identified. Some vaccine candidates are now undergoing clinical trials to test their safety and effectiveness in humans. Furthermore, since most clinical vaccine trials are conducted in naive people, it will be critical to expand these studies to discover if individuals who have already been infected with dengue can be efficiently and safely vaccinated.

On the other hand, the need to develop well-protective antiviral properties with moderate toxicity, low likelihood of viral resistance, and adequate stability to ensure absorption and dispersion has stymied antiviral research against dengue. Hence, continuous advancements in screening methodologies, X-ray modeling, and easily accessible databases provide a promising foundation for the discovery of novel therapies, but additional examination of existing medications is also required. Combination treatment appears to be the best antiviral method for addressing the current anti-DENV medication development roadblocks.

## Figures and Tables

**Table 1:** DENV vaccines currently under development.

Vaccine Type	Vaccine Name	Developer	Current Stage	Target Antigen	Strategy	Key Clinical Outcome	References
Live attenuated	Dengvaxia© (CYD-TDV)	Sanofi Pasteur	Licensed	Live virus	DENV 1–4 genes substituted for the YF17D virus genes (prM/E).	Age limit; increased risk of severe dengue in seronegative subjects but high effectiveness and safe in seropositive individuals	[11,17,18]
	Tetravax; TV003/TV005	NIH (USA); Butantan Institute (Brazil); Panacea Biotec Ltd. (India)	Phase II/III	Live virus	Attenuation of DENV1, DENV3, DENV4, and a DENV2/DENV4 chimerical by excluding 30 nucleotides from the 3′ UTR.	Well-tolerated; balanced immune response in subjects, effective with administration of a single dose. Adverse reaction (mild rash)	[19,20]
	TAK-003; DENVax	Mahidol University; Inviragen; Takeda	Phase III	Live virus	DENV2 PDK-53 attenuated vaccine coding sequences are replaced with DENV1, DENV3, and DENV4 coding sequences.	Immunogenic and well-tolerated in multiple phase I and II clinical studies, independent of the participants’ age or serostatus, safety profile not entirely known	[21]
	TDEN F17/F19	WRAIR and GSK	Phase II	Live virus	Involving primary cells of dog kidney (PDK) and lung cells of fetal rhesus (FrhL) in serial passages	Proven to be a safe, well-tolerated, and immunogenic DENV vaccine candidate in phase II trial	[22,23]
Inactivated	TDEV-PIV	GSK, Firocruz and WRAIR Merck	Phase I	Inactive virus	Employing adjuvants and purified formalin-inactivated virus	Well-tolerated, immunogenic in naive and seropositive individuals. No risk of re-activation and good immuno-logical balance	[24,25]
Recombinant subunit	V180	GSK, Firocruz and WRAIR Merck	Phase I/II	80% of the E protein	DEN-80E-containing recombinant truncated protein	Induce steady immune responses against all DENV serotypes, decreasing the likelihood of the ADE effect	[26,27]
Nucleic acid (DNA)	TVDV	U.S Naval Medical Research Centre	Phase I	prM and E proteins	prM/E proteins are encoded via a recombinant plasmid vector	Stable but lack of immunogenicity. Plasmid modification required.	[28,29]
	D1ME100	US Naval Medical Research Center	Phase I	prM and E proteins	recombinant plasmid vector encoding prM/E	No neutralizing antibody response detected in individuals with low-dose immunization	[30]

**Table 2:** Summary of dengue potent inhibitors based on their target.

Target	Antiviral Name	Mechanism of Antiviral Action	Method	Results	Reference
E protein	MLH40	Inhibit virus entry	DENV inhibition assays, molecular docking	IC50 (µM): 24–31, Docking score (kcal/mol): −29.42	[50]
	DET4	Inhibit virus entry and binding	Molecular docking, molecular dynamics	Docking score (kcal/mol): −9.6	[51]
	BP34610	Inhibit virus entry	HTS, cell-based assay	EC50 (µM): 0.48 ± 0.06, CC50 (µM): 94.55 ± 1.77	[52]
C protein	Pep14–23	Inhibit interaction of C protein lipid droplets	Molecular docking	Docking score (kcal/mol): 5.30 (± 0.96)	[53]
	VGTI-A3/VGTI-A3-03	Inhibit capsid protein	Cell-based assay	IC50 (µM); VGTI-A3: 0.11, VGTI-A3-03: 0.025, CC50 (µM); VGTI-A3: >50, VGTI-A3-03: 11.6	[54]
NS2B/NS3 pro	Nelfinavir	Inhibit protease enzyme	Molecular modeling, cell-based assay, yield-reduction assay	EC50 (µM): 3.5 ± 0.4	[55]
	Diaryl(thio)ethers (Compound **7** and **8**)	Inhibit protease enzyme	Cell-based assay	IC50 (µM): Compound **7**: 9.3, Compound **8**: > 3, EC50 (µM): Compound **7**: 2.5, Compound **8**: > 3	[56,57]
	MB21	Inhibit protease enzyme	Protease inhibition assay, cell-based assay	IC50 (µM): 5.95	[56]
	Policresulen	Inhibit protease enzyme and destabilization	Cell-based assay	IC50 (µM): 4.99	[57]
	Compound **45a**	Inhibit protease enzyme	Cell-based assay	IC50 (µM): 0.26 ± 0.03	[58]
	Compound **104**	Inhibit protease enzyme	Cell-based assay	IC50 (µM): 0.557 EC50 (µM): 3.4, CC50 (µM): > 100	[59]
	Compound **14**	Inhibit protease enzyme	Molecular docking, Protease inhibition assay, cell-based flavivirus immune detection, cell viability assay	Docking score (kcal/mol): −10.86, IC50 (µM): 6.7 ± 1.1, EC50 (µM) (BHK21):5.0 ± 1.1, CC50 (µM) (BHK21: > 500; EC50 (µM) (HuH7): 5.0 ± 0.2, CC50 ((µM) HuH7): 55	[60]
	Compound **C**	Inhibit protease enzyme	HTS, Molecular modeling, protease inhibition, cell-based assay	EC50 (µM): 8.97 ± 0.05, CC50 (µM): 76.11	[61]
	Carnosine	Inhibit protease enzyme	Protease assay, molecular docking, cell-based assay	EC50 (µM): 52.3, IC50 (µM): 63.7	[62]
	A1-A5	Inhibit protease enzyme	Molecular docking	Docking score (kcal/mol); A1: −10.86, A2: −11.07, A3: −10.97, A4: −10.71, A5: −10.33	[63]
	8 g and 8 h	Inhibit protease enzyme	Protease activity assay, protease inhibition assay, molecular docking	IC50 (µM); 8 g: 13.9 ± 1.4, 8 h: 15.1 ± 1.3; Docking score (kcal/mol); 8 g: −8.8, 8 h: −8.8	[64]
	Luteolin	Inhibit protease enzyme	Molecular docking	Docking score (kcal/mol): −7.7	[65]
	Hesperetin	Inhibit protease enzyme	Protease assay activity, cell-based assay, molecular docking	EC50 (µM): 326.4, Docking score (kcal/mol): −7.2	[66]
	Epigallocatchin	Inhibit protease enzyme	Molecular docking	Docking score (kcal/mol): −13.29	[67]
	CC 3	Inhibit protease enzyme	Protease assay activity, cell-based assay	IC50 (µM): 39.17 ± 6.69 EC50 (µM): 2.68 ± 0.64 CC50 (µM): 32.34 ± 4.72	[68]
	4-hydroxy-6-(9,13,17-trimethyldodeca- 8,12,16-trienyl)2(3 H)-benzofuranone	Inhibit protease enzyme	Protease assay activity, cell-based assay	IC50 (µM): 403.14 ± 33.03	[69]
	Dryobalanops aromatic	Inhibit protease enzyme	Protease assay activity	IC50(μg/mL): 0.30 ± 0.16	[70]
	Diasarone-I	Inhibit protease enzyme	Cell-based assay, molecular docking	EC50 (µM): 4.5 uM CC50(µM): > 80, Docking score (kcal/mol): −7.2	[71]
	Isobiflorin	Inhibit protease enzyme	Protease assay activity	IC50 (µM): 58.9 ± 1.3	[72]
	Compound **1**	Inhibit protease enzyme	Protease inhibition assay, cell viability assay, western blot, RT-PCR, IF microscopy	IC50 (µM); 7.1, CC50 (µM): 35.4	[73]
NS4A	Compound-**B**	Inhibit viral replication	Cell-based assay	IC50(µM): 1.81 ± 0.15 (DENV1), 1.32 ± 0.19 (DENV2), 2.66 ± 0.08 (DENV3), 4.12 ± 0.15 (DENV4), CC50 (µM): 13.5 ± 3.1	[74]
NS4B	AM404	Inhibit NS4B	Cell-based assay	EC50 (µM): 2.2	[75]
	NITD-688	Inhibit NS4B	HTS, in vivo study	1.44-log viremia reduction (3 days), 1.16-log viremia reduction (48 h)	[76]
	Compound **14a**	Inhibit NS4B	Cell-based assay	IC50 (µM): > 30	[77,78]
NS5 RdRP	2′-C-methylcytidine	Inhibit viral replication	Cell-based assay	IC50 (µM): 19.3 ± 0.7	[77]
NS5 MTase	Azidothymidine-based triazoles (9a,11a,11b,11i,15i,17b,19b)	Inhibit viral RNA capping	Cell-based assay, molecular modeling	IC50 (µM); 9a: 21 ± 7.8 11a: 7.9 ± 0.7 11b: 31 ± 1.4 11i: 22 ± 7.0 15i: 22 ± 4.9 17b: 14 ± 4.2 19b: 23 ± 4.9; EC50(µM); 9a: 8.4 ± 0.8 11a: 7.3 ± 1.0 11b: 14 ± 2.8 11i: 7.5 ± 0.7 15i: 9.6 ± 0.6 17b: 7.4 ± 1.4 19b: 11 ± 0	[78]
	BG-323	Inhibit viral RNA capping	Cell-based assay	IC50 (µM): 7.3± 2.9, EC50 (µM): 8.5 ± 2.1, CC50 (µM): 184.0± 41.7	[79,80]
	NSC 12155	Inhibit viral RNA capping	Molecular docking, cell-based assay	IC50(µM): 1.4, EC50(µM): 1.0, CC50(µM): 49 Docking score (kcal/mol): 7.2 kcal/mol	[81]

## Data Availability

Not applicable.
